# Retrospective Reports of Developmental Stressors, Syndemics, and Their Association with Sexual Risk Outcomes Among Gay Men

**DOI:** 10.1007/s10508-015-0479-3

**Published:** 2015-06-19

**Authors:** Tyler G. Tulloch, Nooshin K. Rotondi, Stanley Ing, Ted Myers, Liviana M. Calzavara, Mona R. Loutfy, Trevor A. Hart

**Affiliations:** 1 Department of Psychology, Ryerson University, 350 Victoria Street, Toronto, ON M5B 2K3 Canada; 2 Dalla Lana School of Public Health, University of Toronto, Toronto, ON Canada; 3 Department of Medicine, University of Toronto, Toronto, ON Canada; 4 Present Address: Musculoskeletal Health and Outcomes Research, Li Ka Shing Knowledge Institute, St. Michael’s Hospital, Toronto, ON Canada

**Keywords:** Minority stress, Syndemics, HIV prevention, Bullying, Childhood abuse, Sexual orientation

## Abstract

Gay and bisexual men (GBM) continue to have a disproportionately higher HIV incidence than any other group in Canada and the United States. This study examined how multiple co-occurring psychosocial problems, also known as a syndemic, contribute to high-risk sexual behavior among GBM. It also examined the impact of early life adversity on high-risk sexual behavior as mediated by syndemic severity. A sample of 239 GBM completed self-report questionnaires at baseline and 6-month follow-up. Syndemic variables included depression, polysubstance use, and intimate partner violence. Early life adversity variables measured retrospectively included physical and verbal bullying by peers and physical and sexual abuse by adults. A Cochran–Armitage trend test revealed a proportionate increase between number of syndemic problems and engagement in high-risk sex (*p* < .0001), thereby supporting syndemic theory. All early life adversity variables were positively correlated with number of syndemic problems. A bootstrap mediation analysis revealed indirect effects of two types of early life adversity on high-risk sex via syndemic severity: verbal bullying by peers and physical abuse by adults. There was also an overall effect of physical bullying by peers on high-risk sexual behavior, but no specific direct or indirect effects were observed. Consistent with syndemic theory, results provide evidence that certain types of early life adversity impact high-risk sex later in life via syndemic problems. Behavioral interventions to reduce sexual risk among GBM should address anti-gay discrimination experienced before adulthood as well as adult psychological problems.

Since the beginning of the human immunodeficiency virus (HIV) pandemic, over 75 million people have been infected and almost 36 million people have died (UNAIDS, 2013). Although the number of new infections has decreased by 33 % since 2001, the number of people living with HIV continues to increase due to improved access to antiretroviral treatment. In Canada and the United States, the dominant mode of transmission in both countries is unprotected anal intercourse between men (Centers for Disease Control and Prevention, 2011; Public Health Agency of Canada, 2013). Moreover, gay and bisexual men (GBM) in Canada and the United States continue to have a disproportionately higher HIV incidence and prevalence than any other group. In Canada, the risk group with the largest proportion of new HIV infections in 2012 was GBM, with 50.3 % of new infections being attributed to sexual contact between men. This trend is similar in the United States, where the number of new infections attributed to sex between men increased between 2008 and 2011 and sex between men was estimated to account for 62.0 % of new infections in 2011.

There is evidence that the disproportionate burden of HIV among GBM can be explained by the high per-act probability of transmission via unprotected receptive anal intercourse (Beyrer et al., 2012). The per-act probability of HIV transmission for the receptive partner is estimated to be 1.4 % if ejaculation occurs inside the rectum and 0.6 % without ejaculation (Jin et al., 2010). Despite education and HIV prevention initiatives aimed at reducing high-risk sexual behavior, a large proportion of GBM continue to engage in unprotected anal intercourse. In a study of 362 GBM from Toronto, 28 % of HIV-positive men and 13 % of HIV-negative men reported engaging in unprotected anal intercourse with a non-regular partner of any HIV status in the past 6 months, representing 19 % of the total sample (Calzavara et al., 2012). In a study of 521 primarily HIV negative (85 %) GBM from Ottawa, 26.3 % reported unprotected insertive anal intercourse with internal ejaculation and 22.5 % reported unprotected receptive anal intercourse with internal ejaculation in the past 2 months with a partner of any HIV status (O’Byrne, Bryan, & Roy, 2013). In a study of 965 GBM from Montréal, 12.2 % reported engaging in unprotected anal intercourse with a non-regular partner of any HIV status during their most recent sexual encounter within the past 2 months (Lambert et al., 2011). Finally, in a study of 1,017 GBM recruited during the 2005 Toronto Gay Pride Festival, approximately 15 % of HIV negative and 38 % of HIV-positive participants reported engaging in unprotected anal intercourse with partners of opposite or unknown HIV status (Hart, James, Hagan, & Boucher, 2010).

These rates are similar to those reported outside of Canada. This frequency is somewhat consistent with a meta-analysis examining the prevalence of unprotected anal intercourse among HIV-positive men who have sex with men in the United States where 26 % of HIV-positive men reported engaging in either insertive or receptive unprotected anal intercourse with partners of opposite or unknown HIV status over time periods ranging from the past 2 weeks to the past year (Crepaz et al., 2009). The frequency of unprotected anal intercourse increased to 43 % with partners of any HIV status. Given that GBM continue to engage in high-risk sexual behavior despite the risk of transmitting or contracting HIV or other sexually transmitted infections, further investigation into psychosocial factors that impact high-risk sexual behavior is warranted (Wolitski & Fenton, 2011).

To date, many theories have further supported the understanding of the underlying factors perpetuating the HIV epidemic among GBM. The term syndemic describes the interaction between two or more diseases or psychosocial problems, which in an additive or synergistic fashion can contribute to a larger disease burden within a given population (Baer, Singer, & Susser, 2003; Singer & Claire, 2003). Syndemics theory conceptualizes the supportive nature of harmful social conditions and social connections within a population, which can assist in the development and sustainability for disease and negative health outcomes, such as the case in HIV (Singer, 2009; Singer & Claire, 2003). This framework was first applied in HIV to a tripartite health condition encompassing substance abuse, violence, and AIDS (SAVA; Singer, 1996). In this preliminary model, SAVA demonstrated the relationship between multiple psychosocial epidemics that mutually exacerbate each other to increase HIV transmission risk.

Several studies have since documented the additive effects of multiple psychosocial problems in predicting high-risk sexual behavior and HIV-positive status among GBM (e.g., Dyer et al., 2012; Ferlatte, Hottes, Trussler, & Marchand, 2013; Halkitis et al., 2013; Jie, Ciyong, Xueqing, Hui, & Lingyao, 2012; Kurtz, Buttram, Surratt, & Stall, 2012; Moeller, Halkitis, & Surrence, 2011; Mustanski, Garofalo, Herrick, & Donenberg, 2007; Parsons, Grov, & Golub, 2012; Stall et al., 2003). These studies have expanded the SAVA model to include other psychosocial problems such as depression and specific types of violence such as intimate partner violence and sexual and physical abuse in childhood and/or adulthood.

The concept of minority stress, defined as psychosocial stress resulting from having minority status (Brooks, 1981), has been incorporated into research examining adverse mental health outcomes among gay men (Meyer, 1995). The Minority Stress Model (Meyer, 2003) adds to the syndemics literature by indicating the nature of the relationships among psychosocial problems. The Minority Stress Model is used to explain why minority group members such as GBM experience disproportionately higher rates of mental health problems as compared with members of non-minority groups. According to this model, stressors such as stigma, prejudice, and discrimination that are directed toward GBM create a hostile social environment resulting in mental health problems. Several studies have offered support for the Minority Stress Model. For example, Lewis, Derlega, Griffin, and Krowinski (2003) reported that life stress and gay-related stress were independently associated with depressive symptoms, and that those individuals reporting higher levels of gay-related stress and stigma consciousness reported greater severity of depressive symptoms. A meta-analysis conducted by Newcomb and Mustanski (2010) demonstrated small to moderate effect sizes for the relation between internalized homophobia and internalizing mental health problems such as depression and anxiety among gay, lesbian, and bisexual individuals. Hatzenbuehler, Nolen-Hoeksema, and Erickson (2008) tested the Minority Stress Model and reported that proximal minority stress within the past 12 months was associated with depression symptoms as well as high-risk sexual behavior, indicating that minority stress may play a role in both mental and sexual health outcomes among GBM.

The syndemics literature has been slow to integrate the concept of minority stress into syndemics models; however, recent studies have begun to incorporate gay-specific victimization into these models (Ferlatte et al., 2013; Herrick et al., 2013). Ferlatte et al. included forms of anti-gay victimization such as lifetime verbal or physical harassment and job discrimination within the syndemics model and found that with every additional type of marginalization reported, there was a greater likelihood of experiencing psychosocial issues such as emotional distress, social isolation, substance use, and mental health problems. The presence of each additional psychosocial issue was in turn associated with a greater likelihood of engaging in unprotected anal intercourse. The results of this study are consistent with the Minority Stress Model and demonstrate that anti-gay experiences are also involved in the HIV syndemic.

Herrick et al. (2013) also included anti-gay victimization within a syndemics model, examining syndemics production from a life-course perspective. This study examined the differential impact of early life adversity (i.e., experienced prior to age 18) and adversity experienced during adulthood on syndemic production among MSM. Early life adversity included physical and sexual abuse, childhood victimization, gay-related victimization, and perceived lack of attainment of masculinity norms among others. Adulthood adversity variables included sexual assault and the experience of overt discrimination such as being fired from a job or refused an apartment. Syndemic variables were substance use, depression, stress, sexual compulsivity, and intimate partner violence. After controlling for sociodemographic information and adversity variables, Herrick et al. found that although past-year adversity had the greatest impact on syndemic production, early life adversity (particularly childhood victimization and perceived lack of masculinity attainment) contributed to the overall model. Although syndemic conditions have been associated with high-risk sexual behavior in prior studies (e.g., Mustanski et al., 2007; Parsons et al., 2012; Stall et al., 2003), high-risk sexual behavior was not examined as an outcome in the Herrick et al. study.

These recent studies (Ferlatte et al., 2013; Herrick et al., 2013) incorporate the concept of minority stress into syndemic theory by demonstrating an association between anti-gay victimization and the presence of multiple co-occurring psychosocial health problems among GBM. More specifically, early life adversity in the form of childhood sexual abuse, physical abuse, and perceived attainment of masculinity norms have been associated with the syndemic condition (Herrick et al., 2013), and lifetime anti-gay victimization, particularly harassment and job discrimination, have been associated with the syndemic condition as well as with unprotected anal intercourse (Ferlatte et al., 2013). To date, no study has examined how childhood adversity is associated with both the syndemic condition and high-risk sexual behavior among GBM. The goals of the current study, therefore, are to examine the syndemic condition in an urban sample of Canadian GBM, and to extend previous research by examining the association between early life adversity and high-risk sexual behavior in adulthood as mediated by the syndemic condition. It is hypothesized that: (1) participants who experience a higher number of psychosocial health problems will be more likely to report engaging in high-risk sexual behavior; and (2) each of the early life adversity variables will be positively associated with high-risk sexual behavior in adulthood, and this association will be mediated by the number of psychosocial health problems experienced in the syndemic condition.

## Method

### Participants

Participants were self-identified men who have sex with men recruited for the Sexual Health and Attitudes Research Project (SHARP), a study conducted in Toronto, Canada from 2006 to 2009 examining health-related attitudes, beliefs, and behaviors of GBM (e.g., James et al., 2012). SHARP participants were recruited from advertisements in local print media aimed toward gay men as well as from the Polaris Seroconversion Cohort Study (Calzavara et al., 2003), a 5-year longitudinal study comparing recently seroconverted GBM and HIV-negative controls. As the Polaris study was designed to recruit equivalent groups of HIV-positive and HIV-negative men, the SHARP sample likewise included equivalent groups of HIV-positive and HIV-negative men. Inclusion criteria were that participants must (1) be men of 18 years of age or older, (2) speak and understand English, (3) have had sexual contact with another male in the past 6 months, and (4) give informed consent to participate in the study. Participants in SHARP were excluded if their ability to understand and complete the study measures was compromised due to physiological or psychological constraints. A total of 302 participants completed the baseline assessment and 239 participants completed the 6-month follow-up assessment, representing an attrition rate of 20.8 %. Sociodemographic characteristics are presented in Table 1.

**Table 1:** Sociodemographic characteristics and descriptive statistics (*n* = 239)

Variable	*n* (*M*)	% (SD)
Ethnicity		
White	182	76.2
Latino, Hispanic	13	5.4
Mixed race, Multiracial	12	5.0
Aboriginal, First Nations	7	2.9
Black	4	1.7
Other	31	12.9
Education		
No high school diploma	21	8.8
High school diploma or GED	26	10.9
<3 years post-secondary	85	35.6
Bachelor’s degree	75	31.4
Graduate or professional degree	31	13.0
Employment^a^		
Unemployed: on disability	59	24.7
Unemployed: other	47	19.7
Student (full- or part-time)	18	7.5
Employed part-time: <40 h	34	14.2
Employed full-time: 40+ h	93	38.9
Income		
Less than $10,000	27	11.3
$10,000 to $19,999	69	28.9
$20,000 to $29,999	19	7.9
$30,000 to $39,999	22	9.2
$40,000 to $49,999	34	14.2
$50,000 or more	58	24.3
Sexual orientation		
Bisexual	13	5.4
Gay or homosexual	223	93.3
Sex role		
Top	50	20.9
Bottom	60	25.1
Versatile	116	48.5
HIV status		
Negative	125	52.3
Positive	114	47.7
Syndemic variables (scored above cut-off)		
Depression	90	37.7
Polysubstance use	48	20.1
Intimate partner violence	39	16.3
Engaged in high-risk sexual behavior	44	18.4
Age^b^	44.2	9.72
Early life adversity^b^		
Verbal peer victimization	23.58	16.28
Physical peer victimization	10.02	43.42
Childhood sexual abuse	9.29	5.64
Childhood physical abuse	9.42	5.20

Percentages may not add up to 100 due to missing data or participants refusing to answer

^a^Some participants reported belonging to more than one employment category

^b^The values presented for *age* and early life adversity variables are mean and standard deviation

### Procedure

Inclusion criteria were assessed via telephone, and after being informed of the purpose for the study, all participants provided informed consent. Study measures were completed on site in the HIV Prevention Laboratory at Ryerson University. All eligible and consenting participants completed self-report measures via Audio Computer-Assisted Self-Interview (A-CASI) at two time points: baseline and 6-month follow-up. A-CASI methodology has been shown to increase reporting of stigmatized behaviors relative to interviewer-administered personal interview (Perlis, Des Jarlais, Friedman, Arasteh, & Turner, 2004; Tourangeau & Smith, 1996; Turner, Ku, Rogers, Lindberg, & Pleck, 1998). Participants were provided $50 compensation after the initial baseline assessment and received an additional $30 compensation after the 6-month follow-up for approximately 3.5 h of time in total. The study received ethics approval from the Ryerson University and University of Toronto research ethics boards.

### Measures

#### Early Life Adversity

Types of early life adversity included verbal and anti-gay physical peer victimization during childhood and physical and sexual abuse by adults. Not all peer victimization was necessarily anti-gay in nature. The physical peer victimization reflects anti-gay bullying whereas the verbal peer victimization and physical and sexual abuse by adults reflects early life adversity unrelated to perceived sexual orientation or gender identity. These variables were measured at baseline. For each variable, continuous scores were used, with higher scores indicating greater victimization.

##### Verbal Peer Victimization

Verbal peer victimization was assessed using the Teasing Questionnaire-Revised (TQ-R; Storch et al., 2004). The TQ-R is a 29-item self-report scale used to measure memories of childhood teasing in adults across five domains: performance, academics, social behavior, family background, and appearance. Participants indicate to what degree they were teased about each topic based on a five-point Likert-type scale ranging from “I was never teased about this” to “I was always teased about this.” The possible total score ranges from 0 to 116. Sample items from the TQ-R include “I was teased about being ugly or unattractive” and “I was teased because of the way that I spoke.”

##### Anti-gay Physical Victimization

Experiences of gay- or bisexual-related physical victimization prior to the age of 18 were assessed using subscales of a self-report scale created by D’Augelli, Pilkington, and Hershberger (2002). The full scale assesses the number of times before and after the age of 18 that participants experienced bullying because they were thought to be gay or bisexual. Types of bullying assessed include being verbally insulted, threatened with physical violence, having an object thrown at them, being punched, kicked, or beaten, threatened with a weapon, forced to have sex, or threatened to have their sexual orientation disclosed against their will. For the purpose of the study, physical bullying refers to being the recipient of the following acts of physical aggression prior to the age of 18: being threatened with physical violence, having an object thrown at them, or being punched, kicked, or beaten. Anti-gay physical victimization was measured as a continuous variable, with the total score representing the number of times participants reported experiencing physical bullying as defined above.

##### Childhood Sexual and Physical Abuse

The Childhood Trauma Questionnaire-Short Form (CTQ-SF; Bernstein et al., 2003) is a 28-item shortened and empirically validated version of the 70-item Childhood Trauma Questionnaire (Bernstein et al., 1994). It is a self-report questionnaire designed to retrospectively assess abuse and neglect experienced during childhood. In previous research, childhood sexual abuse and physical abuse were the primary forms of abuse included in syndemics models (e.g., Herrick et al., 2013; Parsons et al., 2012), and were thus a focus of the present study. For the CTQ-SF, participants were presented with statements regarding experiences of abuse and asked to indicate how true each statement was of their experience growing up based on a five-point Likert-type scale ranging from “never true” to “very often true.” The possible total score for the each of the sexual and physical abuse subscales ranges from 5 to 25. Sample items from the CTQ-SF sexual and physical abuse subscales, respectively, include “When I was growing up, someone molested me” and “When I was growing up, people in my family hit me so hard that it left me with bruises or marks.”

#### Syndemic Variables

Psychosocial problems included in the syndemic variable (mediator) were depression, polysubstance use, and intimate partner violence. These variables were assessed at baseline, and therefore temporally preceded the outcome variable of high-risk sexual behavior that was assessed at 6-month follow-up. For each variable, cut-off scores were used in order to identify whether or not participants experienced any given psychosocial problem.

##### Depression

Depression was measured using the Center for Epidemiological Studies-Depression Scale (CES-D; Radloff, 1977), a 20-item self-report scale designed to measure past-week depression symptoms in the general population. Participants rated how often they experienced each symptom over the past week on a four-point Likert-type scale ranging from “rarely or none of the time (less than 1 day)” to “most or all of the time (5-7 days).” Depression was dichotomized into depressed or not according to clinical cut-offs from previous research studies (Beekman et al., 1997; Radloff, 1977) which indicate that a total score of 16 or above is indicative of depressive symptomatology.

##### Polysubstance Use

Participants were asked about their frequency of drug use over the past 6 months. Drugs included marijuana, heroin, cocaine, speedball, methamphetamine (crystal meth), ecstasy, gamma-hydroxybutyric acid (GHB), amphetamines (including speed or other uppers), ketamine (K), and barbiturates. Participants who reported using three or more of these drugs at least once each during the past 6 months were considered to have engaged in polysubstance use.

##### Intimate Partner Violence

Participants indicated whether they had ever been hit by a primary or casual sex partner. Participants who responded “yes” to being hit by either type of partner were considered to have experienced intimate partner violence.

##### Syndemic Count Variable

A syndemic count variable was created to indicate the number of psychosocial problems experienced by each participant. As only six participants experienced all three psychosocial problems, this count variable ranged from “0” to “2-3.” This syndemic count variable was used as the mediating variable for the analyses of indirect effects.

#### High-Risk Sexual Behavior

Participants were considered to have engaged in high-risk sexual behavior if they reported having unprotected anal intercourse, either insertive or receptive, with a male partner of opposite or unknown HIV serostatus in the past 6 months (dichotomized, yes vs. no). High-risk sexual behavior was assessed at the 6-month follow-up assessment, and was therefore preceded temporally by the syndemic variable.

### Statistical Analyses

Descriptive analyses were performed using SAS 9.3 (SAS Institute, 2011) to determine the prevalence of early life adversity variables, the syndemic condition and high-risk sexual behavior. The Cochran–Armitage test was used to evaluate the trend between the syndemic count variable and high-risk sexual behavior. A series of logistic regressions were used to examine the odds ratios and confidence intervals associated with engaging in high-risk sexual behavior based on the syndemic count variable (i.e., comparing 0 vs. 1 psychosocial problem, 0 vs. 2–3 psychosocial problems, and 1 vs. 2–3 psychosocial problems).

To estimate indirect effects, four mediation models were evaluated in the overall sample using the bootstrapping method (Preacher & Hayes, 2004). Specifically, the syndemic condition was evaluated as mediating the association between each of the four early life adversity variables and high-risk sexual behavior in separate models, while controlling for age and the remaining three variables measuring early life adversity. A separate mediation was conducted for each of the four early life adversity variables while controlling for the remaining three early life adversity variables. Mediation analyses involved the nonparametric re-sampling of the dataset to make repeated estimates (10,000 times) using the PROCESS macro in SAS 9.3. The validity of mediation testing using a dichotomous-dependent variable with bootstrapping is supported by Hayes and Preacher (2010). This approach is more powerful than the three-step regression method of Baron and Kenny (1986) and the Sobel test (Sobel, 1982) because it minimizes the number of statistical tests used, quantifies the mediation effects, and makes no assumptions about the shape of the distribution for the indirect effect (Hayes & Preacher, 2010). Moderated mediation models were also explored using the procedures recommended by Preacher, Rucker, and Hayes (2007). To investigate the potential moderating effects of HIV status, we evaluated whether the size of the mediated path differed significantly among HIV-positive and HIV-negative groups in each of the four models.

## Results

The demographic profile of this sample is provided in Table 1. The participants were predominantly middle-aged, White, had at least some post-secondary education, and were employed full- or part-time. Descriptive statistics of the early life adversity, syndemic, and high-risk sexual behavior variables are also presented in Table 1. The frequency of high-risk sexual behavior reported in the total sample was 18.4 %, including 13.6 and 23.7 % of HIV-negative and HIV-positive participants, respectively. A series of independent samples *t* tests revealed that dropouts and completers did not differ on any of the early life adversity variables. A series of *χ* ^2^ tests revealed that dropouts and completers did not differ on any of the syndemic or sociodemographic variables except for sexual orientation, *χ* ^2^(2) = 11.76, *p* = .003, and total annual income, *χ* ^2^(5) = 12.03, *p* = .034, with dropouts being more likely to identify as straight or bisexual and to report belonging to a lower annual income category. A logistic regression indicated that there were no associations between either sexual orientation or annual income and the outcome variable of high-risk sexual behavior.

The proportion of participants who did not experience any psychosocial health problems, i.e., syndemic conditions, was 46.9 % (*n* = 112), whereas 34.7 % (*n* = 83) experienced one condition, 15.9 % (*n* = 38) experienced two conditions, and 2.5 % (*n* = 6) experienced three conditions. A series of binary logistic regressions revealed that all syndemic conditions were significantly associated with one another, such that individuals experiencing one syndemic condition were at greater risk of experiencing any one of the other conditions. The greatest association was found between intimate partner violence and polysubstance use (unadjusted OR 2.36, 95 % CI 1.10–5.04), followed by the association between intimate partner violence and depression (unadjusted OR 2.22, 95 % CI 1.11–4.43). The association between polysubstance use and depression (unadjusted OR 1.53, 95 % CI 0.81–2.90) was not statistically significant; however, the effect size was positive and the CI provides a narrow range of estimates around the point estimate.

**Fig. 1:**
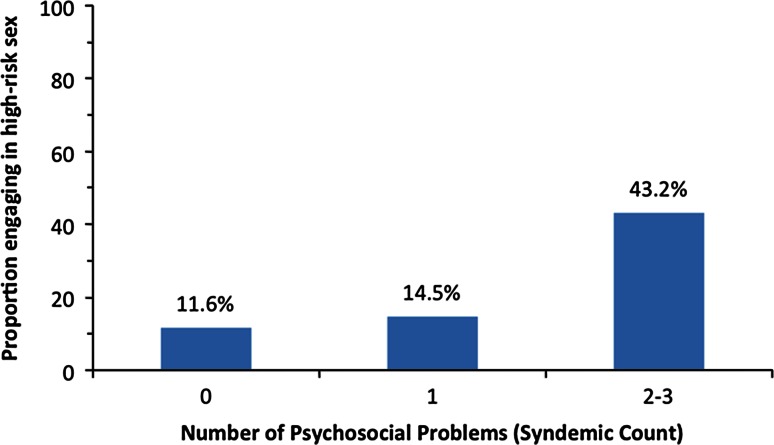
Trend in association between number of psychosocial health issues (syndemic condition) and high-risk sexual behavior, *p* < .0001

Furthermore, a Cochran–Armitage trend test revealed that greater numbers of syndemic conditions were significantly and positively associated with high-risk sexual behavior (*p* < .0001). As shown in Fig. 1, the proportion of individuals engaging in high-risk sexual behavior increases with higher numbers of psychosocial issues. A logistic regression further revealed that relative to individuals without any psychosocial problems, individuals with one problem were 1.29 times more likely to engage in high-risk behavior (95 % CI 0.56–2.99) and those with two to three problems were 5.79 times more likely (CI 2.52–13.28). Relative to individuals with one psychosocial problem, those with two to three problems were 4.50 times more likely to engage in high-risk sexual behavior (95 % CI 1.91–10.57). Although the difference in high-risk sexual behavior between people with zero psychosocial problems versus one psychosocial problem did not reach statistical significance, all other differences were statistically significant (*p* < .05).

### Mediation Analyses

**Fig. 2:**
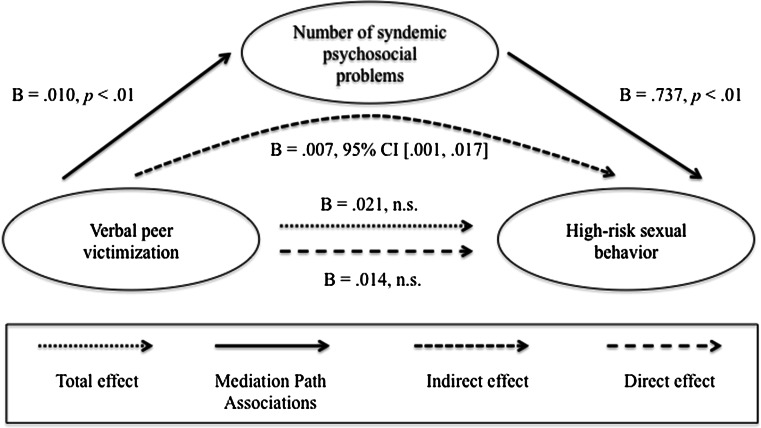
Verbal peer victimization. Results of a mediation analysis controlling for age, physical peer victimization during youth, childhood sexual abuse, and childhood physical abuse

**Fig. 3:**
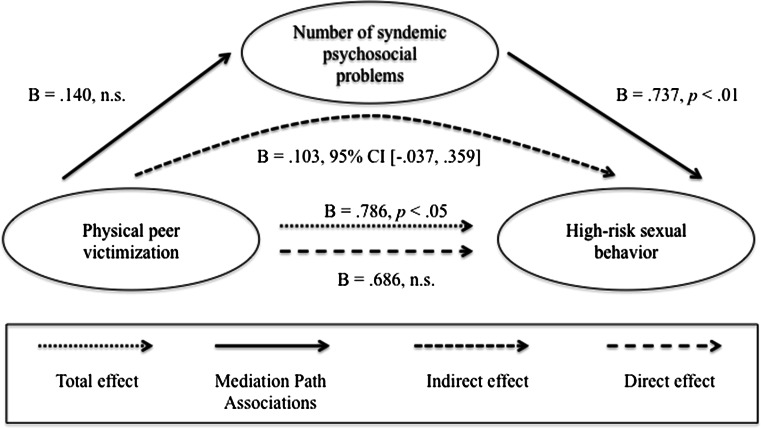
Physical peer victimization. Results of a mediation analysis controlling for age, verbal peer victimization during youth, childhood sexual abuse, and childhood physical abuse

**Fig. 4:**
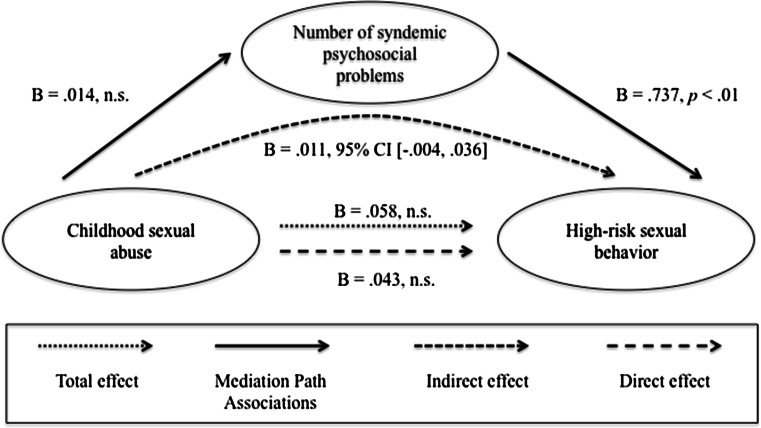
Childhood sexual abuse. Results of a mediation analysis controlling for age, verbal peer victimization during youth, physical peer victimization during youth, and childhood physical abuse

**Fig. 5:**
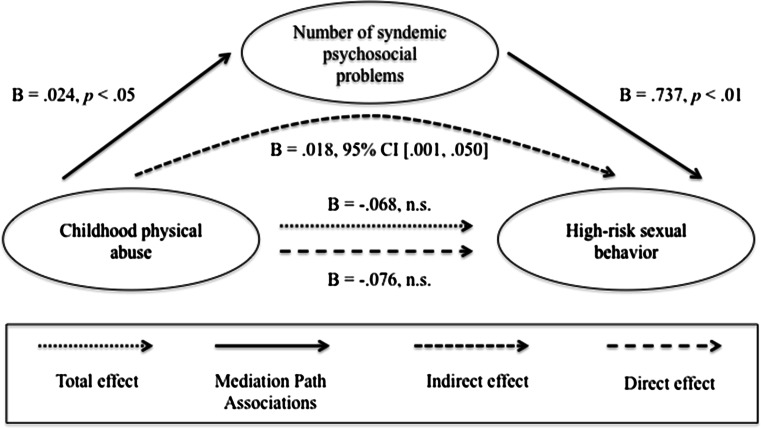
Childhood physical abuse. Results of a mediation analysis controlling for age, verbal peer victimization during youth, physical peer victimization during youth, and childhood sexual abuse

In terms of the moderated mediation models, we found that the direct and indirect effects were not moderated by HIV status (data not shown). Therefore, we focused on estimating four simple mediation models. As mentioned above, only one predictor could be used in each analysis, so in order to examine the impact of each of the four early life adversity variables, four different analyses were conducted with one variable entered as a predictor and the other three entered as covariates. Each of these models accounted for 19 % of the variance in high-risk sexual behavior (*R* ^2^ = 0.19, *p* < .001). Results of the mediation analysis are summarized in Figs. 2, 3, 4, and 5. The direct effect between verbal victimization and high-risk sexual behavior was not significant prior to controlling for the syndemic condition (*B* = .014, *p* = .268). Because an indirect effect of a mediator on the relationship between two variables can still occur when there is no significant direct effect prior to controlling for the mediator (Preacher & Hayes, 2004), indirect effects were also examined. We found a significant indirect effect via the syndemic condition for the relationship between verbal victimization and high-risk sexual behavior (estimated indirect effect: *ab* = .007, 95 % CI 0.001–0.017). In addition, there was a significant total effect between gay- or bisexual-related physical victimization and high-risk sexual behavior (*B* = .786, *p* = .042). However, there was no evidence that the syndemic condition was a significant mediator of the relationship (estimated indirect effect: *ab* = .103, 95 % CI −0.037 to 0.359).

Similarly, the relationship between childhood physical abuse and high-risk sexual behavior was mediated by the syndemic condition (estimated indirect effect: *ab* = .018, 95 % CI 0.001–0.050). There was no significant direct effect of childhood physical abuse on high-risk sexual behavior prior to controlling for the syndemic condition (*B* = −.067, *p* = .151). There were no total, direct or indirect effects between childhood sexual abuse and high-risk sexual behavior.

## Discussion

The present study extends previous research by demonstrating that the number of adult psychosocial health problems experienced by GBM may mediate the relation between early life adversity experienced in childhood and adult risky sex, defined as unprotected anal intercourse with serodiscordant or unknown status partners. Specifically, we found that number of syndemic problems mediated the relationship between childhood physical abuse and high-risk sexual behavior, and between verbal victimization and high-risk sexual behavior among GBM. The study therefore provides support for the syndemic first delineated by Singer (1996) and demonstrates a link between early life adversity, psychosocial problems, and the HIV epidemic among GBM.

Previous research, which provided important data on the association between syndemics and risky sex, was based largely upon cross-sectional data (Dyer et al., 2012; Ferlatte et al., 2013; Halkitis et al., 2013; Jie et al., 2012; Kurtz et al., 2012; Mustanski et al., 2007; Parsons et al., 2012; Stall et al., 2003). For example, Ferlatte et al.’s (2013) recent Canadian study used cross-sectional data and found that with every additional type of marginalization reported, there was a greater likelihood of experiencing psychosocial problems. In this sample of both HIV-positive and HIV-negative GBM, we found that number of syndemic problems at baseline predicted risky sex 6 months later. This effect was strong, and may be at least a linear effect, as only 11.61 % of participants with zero adult syndemic problems engaged in risky sex, versus 14.46 % of participants with one syndemic problem, and 43.18 % of participants with two or three syndemic problems. Although the present study did not assess for effects beyond linear effects, the data suggest that number of psychosocial problems may exert a quadratic effect on subsequent risky sex among GBM.

The early life adversity of verbal harassment, anti-gay physical victimization, sexual abuse, and physical abuse accounted for 19 % of the variance in number of psychosocial health problems. In addition, number of psychosocial problems was the strongest predictor of risky sex in the full model, above and beyond developmental stressors. Given that only 19 % of risky sex was predicted by early life adversities and the psychosocial health problems assessed in the model, it is possible that addition of other psychosocial health problems would have increased the power of this model. For example, recent research has explored the role of sexual compulsivity as a part of the gay men’s HIV syndemic (e.g., Parsons et al., 2012). Sexual compulsivity may be especially important due to its associations with identity as a “barebacker,” that is, someone who intentionally engages in unprotected anal sex (Grov, Parsons, & Bimbi, 2010).

Looking at early life adversity, it was surprising that childhood sexual abuse was not a predictor of sexual risk behavior, given that previous studies have found an association between these two variables (Stall et al., 2003). However, it should be noted that not all studies of GBM have found childhood sexual abuse to be associated with sexual risk behavior, including studies with larger sample sizes (e.g., *n* = 669; Parsons et al., 2012). It is possible that our measure, the CTQ, did not adequately capture the use of force during sex that has previously been associated with sexual risk behavior among GBM in previous studies (e.g., Stall et al., 2003) and one meta-analysis (Arreola, Neilands, Pollack, Paul, & Catania, 2008). It is also possible that childhood sexual abuse exerts its effects via variables not assessed in the present study, such as having emotional numbing when being touched by a man (e.g., O’Cleirigh, Safren, & Mayer, 2012).

Syndemic theory and minority stress theory have both provided important contributions to our understanding of how psychosocial health problems predict risky sex and the HIV epidemic. Future research should consider extensions of these models, such as the Psychological Mediation Framework (Hatzenbuehler, 2009). This model explains that external stressors, such as anti-gay victimization, have their effects both by increasing internal stressors that are unique to sexual minority individuals, such as internalized homophobia, and also by increasing maladaptive psychological processes that can be experienced by all individuals regardless of sexual orientation, such as poor emotional regulation and social isolation. Given that syndemic theory and minority stress theory have been linked in recent research (Ferlatte et al., 2013; Herrick et al., 2013), it is likely that examining the general and gay-specific psychological processes may explain how minority stressors in childhood and adulthood exert their effects on syndemic psychosocial problems among GBM.

### Limitations and Future Directions

The present study, while having a two time-point design, remains a study of adults who retrospectively reported early life adversity. The study is therefore limited by the use of retrospective self-report, which may be subject to biases such as social desirability. The sample was also largely middle-aged, employed, and White, which limits the generalizability of findings for other populations of GBM and other men who have sex with men, including individuals from populations who are at heightened vulnerability for HIV, such as African American or Latino men who have sex with men residing in the United States or Canada (Centers for Disease Control and Prevention, 2012; Public Health Agency of Canada, 2013).

A truly longitudinal study, starting in childhood and continuing in adolescence and adulthood would further strengthen the growing support for syndemic theory and the Minority Stress Model. It may be particularly useful to examine the effect of distal stressors, defined by Meyer (1995, 2003) as external stressors such as anti-gay violence and discrimination, across the lifespan. Although it could be argued that the tie between anti-gay victimization and psychosocial health problems would be strongest if assessed at the same time point, it is also possible that anti-gay victimization might exert more deleterious effects if experienced at a more vulnerable developmental period, such as during the period when first engaging in sexual behavior with a person of the same sex, or if the anti-gay victimization comes at a time of severe family dysfunction or conflict. These more vulnerable periods are termed “sensitive periods” (Gee, Walsemann, & Brondolo, 2012), which are periods that may lead to later dysfunction, especially during life transitions (Pearlin, Schieman, Fazio, & Meersman, 2005), such as transitioning to economic independence in adulthood or the coming out process for sexual minority populations.

It should also be noted that this study and others show that the alleviation of minority stressors and syndemic conditions ultimately rest on changing unhealthy social structures that delegitimize GBM. Both the general literature, which has focused on important inequities due to racism and economic marginalization (e.g., Gee et al., 2012; Pearlin et al., 2005; Singer, 1996; 2009), and the literature on GBM (e.g., Meyer, 1995, 2003; Stall et al., 2003) emphasize the need not only to alleviate stressors experienced by minority individuals, but also to improve equitable access to health care and education, and to alter systems that lead to increased victimization of marginalized populations.

### Implications for Sexual Health Interventions Using a Syndemic Approach

Syndemic researchers have argued that interventions to reduce HIV risk behavior among GBM must pay close attention to the role of syndemic psychosocial problems as potential causes of sexual risk behavior that go beyond a simple lack of safer sex behavioral skills (e.g., Ferlatte et al., 2013). The current findings suggest that early life adversity must be accounted for in syndemic-focused interventions. These interventions need not be focused on gaining insight into early life experiences, but should address the role of early life adversity in creating syndemic conditions. For example, someone who has experienced childhood verbal victimization may also be more likely to have a negative self-image, and may therefore become depressed. In turn, depressed mood among gay men is associated with use of substances to manage negative moods (Carrico et al., 2012). Evidence suggests that drinking and drug use problems may occur in adolescence and young adulthood and may already be associated with sexual risk behavior and poor mental health in this age group (Mustanski, Andrews, Herrick, Stall, & Schnarrs, 2014; Mustanski et al., 2007), suggesting a benefit in addressing the additive effects of abuse and victimization for gay and bisexual male youth. Cognitive-behavioral approaches that address maladaptive cognitions appear promising in alleviating psychological distress related to previous victimization and anti-gay early life experiences (e.g., Hart, Tulloch, & O’Cleirigh, 2014; Satterfield & Crabb, 2010), and so may also be appropriate for syndemic-focused HIV prevention and sexual health promotion interventions for GBM. For example, anti-gay bullying and victimization in childhood may lead to high social anxiety, and social anxiety may lead to increased use of substances to manage sexual relationships (e.g., Roberts, Schwartz, & Hart, 2011; Terlecki & Buckner, 2015). Given that social anxiety is associated with high-risk sexual behavior (Hart & Heimberg, 2005; Hart, James, Purcell, & Farber, 2008), cognitive-behavioral interventions that treat social anxiety may also treat fears of rejection, substance use in sexual situations, and subsequent high-risk sexual behavior (Hart et al., 2014).

In summary, the present study found that a higher number of adult psychosocial problems reported at baseline mediated the relation between childhood verbal victimization and high-risk sexual behavior 6 months later. A similar pattern was found for childhood physical abuse. Interventions to promote sexual health for GBM should address not only adult psychosocial problems, but also early life adversity that may still have an effect on sexual risk behavior for GBM.
